# First results of the application of the Latin Questionnaire in comparison with populations of reference workers obtained through preliminary epidemiological studies

**DOI:** 10.47626/1679-4435-2023-1115

**Published:** 2023-11-24

**Authors:** Daniela Colombini, Olga Menoni, Mirko Pezoa Villanueva, Aquiles Hernandez

**Affiliations:** 1 Scientific Association EPMIES Ergonomics of Posture and Movement International Ergonomics School, Ergonomics, Milán, Italia; 2 Ergonomic Unit, Fondazione IRCCS Ca’ Granda Ospedale Maggiore Policlinico, Ergonomics, Milán, Italia; 3 Consultancy in Occupational Medicine, Ergonomics, Lima, Peru; 4 Cenea, Ergonomics, Barcelona, España

**Keywords:** health surveillance, patient health questionnaire, musculoskeletal system, musculoskeletal diseases, epidemiology, descriptive, vigilancia sanitaria, cuestionario de salud del paciente, sistema musculoesquelético, enfermedades musculoesqueléticas, epidemiología descriptiva

## Abstract

**Introduction:**

The authors proposed an application study of the Latin Questionnaire, an updated protocol to conduct the anamnestic study of work-related musculoskeletal disorders through closed questions and the introduction of a predetermined severity threshold that allows for epidemiological studies to be conducted, comparing the results of the exposed population with those of a reference population. Background: Similar protocols describing work-related musculoskeletal disorders occurring in the previous 12 months are available in the literature. For many of these, problems arise when the results must be processed collectively.

**Objectives:**

Here we present application examples, with comments on the results in terms of statistical significance of the comparison.

**Methods:**

The anamnestic study of the Latin Questionnaire is based on symptoms: discomfort, pain, and evaluation of paresthesia. Each symptom is described considering: location, duration, number of episodes, irradiation, and treatment. The model, which covers the previous 12 months, is designed to identify in the spine, upper and lower limbs: positive anamnestic case, case with minor disorders, negative case.

**Results:**

The original application examples show the scope of the disorders presented in the groups of workers exposed to known risk, in comparison with those of the reference group: the significance of the differences is estimated statistically.

**Conclusions:**

The Latin Questionnaire, also implemented in the digital format (free to download), allows for comparing the data of exposed and unexposed workers and their statistical significance easily and automatically.

## Abstract (es)

**Introducción:**

Los autores propusieron un estudio de aplicación del Cuestionario Latino, un protocolo actualizado para realizar el estudio anamnésico de los trastornos musculoesqueléticos relacionados con el trabajo a través de preguntas cerradas y la introducción de un umbral de gravedad predeterminada que permiten realizar estudios epidemiológicos comparando los resultados de la población expuesta con los de una población de referencia. Antecedentes: Se encuentran disponibles en la literatura protocolos similares que describen los trastornos musculoesqueléticos relacionados con el trabajo ocurridos en los 12 meses anteriores. Para muchos de estos surgen problemas cuando los resultados deben procesarse colectivamente.

**Objetivos:**

Aquí presentamos ejemplos de aplicación, con comentarios sobre los resultados en términos de significación estadística de la comparación.

**Métodos:**

El estudio anamnésico del Cuestionario Latino se basa en los síntomas: molestia, dolor y evaluación de parestesia. Cada síntoma se describe considerando: localización, duración, número de episodios, irradiación, tratamiento. El modelo, que cubre los 12 meses anteriores, está diseñado para identificar en la columna y en los miembros superiores e inferiores: caso anamnésico positivo, caso con trastornos menores, caso negativo.

**Resultados:**

Los ejemplos originales de aplicación muestran el alcance de los trastornos presentados en los grupos de trabajadores expuestos a un riesgo conocido, en comparación con los del grupo de referencia; la significancia de las diferencias se estima estadísticamente.

**Conclusiones:**

El Cuestionario Latino, implementado también en forma digital (de descarga gratuita), permite comparar de manera fácil y automática los datos de trabajadores expuestos y no expuestos y su significación estadística.

## INTRODUCTION

In order to facilitate the work of the occupational health physicians in prioritization for the conduction of clinical examinations to be carried out on workers exposed to biomechanical overload, the team of Scientific Association EPMIES (Ergonomics of Posture and Movements International Ergonomics School) have developed a number of health surveillance methods have been developed since 1985 for the study and management of occupationally relevant musculoskeletal diseases.^1-5^

This article is a continuation of the article related with the Latin Questionnare.^6^ The first article, recently published in this journal, analytically described the methodological aspects. The objective of this second article, after making a brief summary of study methodology, is to present the results of original epidemiological studies never published.

A working group composed of 37 physicians from 14 Latin American countries participated updating and validating this model (hence named Latin Questionnaire), assessing its intraand inter-rater reliability.^6^

Therefore, the results are related to the initial step in the health surveillance process, the anamnestic phase, which is, however, extremely important because it can provide the company’s physician with:

- A very useful filtering tool for deciding which workers need the second step in the health surveillance process: clinical and instrumental testing;
- A preliminary epidemiological investigation tool for recording the initial collective impact on the health of workers exposed to occupational biochemical overload versus unexposed workers;
- A useful tool if musculoskeletal problems are reported and risk assessment does not show risks due to biomechanical overload. In this case, the preliminary epidemiological study answers the following question: Is there a problem?

These applications have become possible especially due to the introduction of a threshold severity level for the musculoskeletal system (the *positive anamnestic threshold*); which makes it possible to better standardize the results, classifying the workers analyzed as *positive anamnestic cases, minor disorder cases*, and definitively “*negative cases*”.

The updated Latin Questionnaire^6^ for epidemiological anamnestic screening of occupational musculoskeletal disorders is mainly a graphic update of previous versions already proposed and published. Compared with previous versions, the Latin Questionnaire only introduces the study of lower limbs. It was also initially applied to large work populations not exposed to biomechanical overload.^2,3,7,8^ The present work used the most recent results of studies on the presence of positive anamnestic cases in unexposed workers.^8-10^

The results of the anamnestic evaluation collected with the Latin Questionnaire are structured so as to make it easier for them to be entered into free Excel spreadsheets or mobile device software for preparation. These tools not only process the data entered but also generate graphs that show the results of the collective epidemiological assessment, comparing exposed workers (based on the ongoing study) with unexposed workers. The present work will present the results of some preliminary epidemiological studies conducted using anamnestic data obtained with the Latin Questionnaire, which were compared with those from unexposed reference populations and analyzed using the results from significance tests.

### OBJECTIVE OF HEALTH SURVEILLANCE AND GENERAL DEFINITION OF WORK-RELATED MUSCULOSKELETAL DISORDERS (WMSDS)

#### Objective of health surveillance programs

Health surveillance programs conducted both for individual workers and working populations as a whole are managed by occupational physicians and focus on disorders and diseases caused by biomechanical overload, primarily for preventive purposes.

Periodic health surveillance programs can be organized on three levels:

- Level 1: programs son generalized, addressing all exposed workers and aiming to reveal *anamnestic cases*. In this level, medical records of individual workers are registered through interviews conducted by skilled health personnel;
- Level 2: clinical examination of subjects who test positive in the anamnestic examination, aiming to clinically detect cases;
- Level 3: instrumental exams (X-rays, ultrasonography, electromyography, etc.) to determine diagnosis.

The model presented herein is dedicated to the first level; aims to guide health care professionals to obtain anamnestic data with greater precision and agility due to the closed structure of questions and to guided interpretation of questions (the anamnestic thresholds) useful to define the workers who will have access to the second and third levels.

#### Work-related musculoskeletal disorders: pathologies to consider

Chart 1A lists upper limb musculoskeletal disorders considered as work related (according to the Italian legislation).

**Chart 1:** List of occupational upper limb pathologies and recurrent conditions involving the spine, according to type, not compatible with work-related exposure to manual lifting of loads

A. Occupational upper limb pathologies		
Diseases of possible occupational origin		Cubital tunnel entrapment syndrome
		Tendinopathy of the distal triceps insertion
		Dupuytren’s contracture
		Guyon’s canal syndrome
		Cervical rib syndrome
Diseases of very probable occupational origin	Shoulder	Rotator cuff tendinitis
		Tendinitis of the long head of the biceps
		Calcific tendinitis
		Bursitis
	Elbow	Lateral and medial epicondylitis
		Olecranon bursitis
	Wrist-Hand	Flexor / extensor tendinitis (wrist-finger)
		de Quervain’s syndrome
		Trigger finger
		Carpal tunnel syndrome
B. Recurrent pathologies involving the spine		
Congenital malformations		Congenital stenosis of cervical medullary canal
		Baastrup’s disease (“kissing spine disease” - development of neoarthrosis between adjacent spinal processes)
		Congenital spondylolisthesis due to spondylolisis
		Scoliosis (> Cobb 20° and torsion 2)
		Schewermann disease (Schmorl’s nodes plus at least one wedge vertebra causing a curve of 40°)
		Sacralization (fully or partially fused or articulated)
		Klippel-Feil syndrome (vertebral synostosis)
Degenerative disease		Severe lumbar disc disease
		Lumbar protrusion with dural sac impingement
		Herniated lumbar disc (protruded, contained, migrated)
		Outcomes of herniated disc reduction
		Degenerative spondylolisthesis
Recurrent symptoms		Recurrent low back pain or caused by newly formed lesions of the bone tissue

Chart 1B summarizes lower back pathologies influenced by biomechanical overload that should be considered when deciding if an individual is allowed to do manual lifting of loads, including patients.^4^

The most frequent work-related diseases affecting the lower limbs involve hips and knees.^11-13^ The relationship with biomechanical risk factors is less clear for the foot and ankle; the only risk factor cited by several authors is frequent use of pedals.^11,12^

### SUMMARY OF THE LATIN QUESTIONNAIRE FOR ANAMNESTIC EVALUATION, VALIDATION TESTING, AND APPLICATION TECHNIQUES

Here, we will be focusing on the anamnestic interview scheme proposed for the Latin Questionnaire^6^ for screening for work-related musculoskeletal disorders (WMSD), primarily relating to the previous 12 months.

The anamnestic questionnaire is designed to generate an accurate patient history, guided by anatomical illustrations and closed questions to help the healthcare operator compiling it to quickly collect the necessary information (by placing an “X” in the boxes provided). Data collection is also facilitated by the fact that virtually the same scheme and criteria (described below) are used for all of the anatomical segments analyzed.

These are the steps to be followed for collecting anamnestic data, for each of the sites considered in the questionnaire:

- Show the subject the picture illustrating the anatomical site;
- Ask the subject where problems have occurred in the last 12 months;
- Ask for more information only about anatomic areas reported as positive for general presence of any disorder (mark others as “negative”).

Once the guided questions focusing on each anatomical area addressed have been answered, it will possible to define whether:

- The subject is a *positive anamnestic case* for that particular segment and side (i.e. is positive for the anamnestic threshold);
- The subject is a *minor disorder case* having not exceeded the threshold.

Only after obtaining this information, ask the subject: how many years the disorders have been present; if they have caused the subject se to take sick leave; whether the subject knows they suffer from any previously diagnosed diseases.

The questionnaire is divided into five main sections; the specific content of each of these sections will now be illustrated.

#### Personal details (Annex 1, section A).

Certain basic personal information is requested, such as name, date of birth (age), gender, company name, department, and length of employment. The date of completion and name of the person administering the questionnaire are also important.

#### Upper limb disorders: the anamnestic investigation model (Annex 1, sections B and C and D)

The recent anamnestic history section includes symptoms that have appeared over the previous 12 months, broken down by joint and divided into two categories: pain (Annex 1, section B) and paresthesia (Annex 1, section C). Presence of pain must be reported separately for each joint of the upper limb, as well as any radiating pain, and whether the pain appears while moving the joint, lifting weights, or also at rest. For the hand, the location of the pain should be indicated on the picture. Pain or paresthesia lasting only a few minutes is not considered for the purposes of determining an anamnestic case (a typical example would be hand pain upon waking due to incorrect sleeping position). The following information must also be included for each upper limb joint: past treatment; clinical tests/instrumental exams performed; and months or years since onset of the condition.

The second group of symptoms (Annex 1, section C) includes paresthesia (pins and needles, tingling, numbness), and whether the symptoms occur during the day or at night. Each health condition is investigated through a set of standard questions, including the number of episodes of pain or paresthesia that have occurred over the last 12 months, and their duration.

The duration and frequency of pain and paresthesia that classify workers as *anamnestic positive cases*^4,5,6,14^ are based on the following criteria: presence of pain or paresthesia lasting at least one week in the last 12 months, or at least one episode of pain or paresthesia per month in the past 12 months.

In the section concerning the past medical history, subjects are asked if, having reported disorders in the last 12 months, they are aware of any previously diagnosed musculoskeletal pathologies (Annex 1, paragraph C3). In order to confirm such existing pathologies, the subject is asked to present the results of the relevant instrumental examinations documenting the pathology reported.

The part concerning the upper limbs ends with two sections: Annex 1, paragraph C4, which includes remarks on possible future treatments to be recommended to the subject, and Annex 1B, section D, which indicates the level of exposure to biomechanical overload, if known.

#### Spinal disorders: the anamnestic investigation model

The anamnestic investigation model includes a part focusing on the spine, divided into three sections: cervical (Annex 1, paragraph E1), dorsal (Annex 1, paragraph E2), and lumbosacral (Annex 1, paragraph E3), using the same rationale for determining the type and duration of disorders.

Here the subject is asked to report any painful episodes and/or discomfort that have occurred over the previous 12 months.

The criteria to classify *anamnestic positive cases are the following*: a disorder is considered to be “positive”, i.e., over the positive threshold, when it meets relevant criteria. In summary, a case is considered as positive if:

- The discomfort, pain, or paresthesia have been almost continuous over the last 12 months; or
- The pain was episodic but significant in terms of frequency and duration (over the last 12 months). The most representative values are 3-4 episodes lasting 3 days: other combinations (10 episodes lasting 1 day; 6 episodes lasting 2 days; 3 episodes lasting 10 days; 2 episodes lasting 30 days) provide additional useful examples. If the pain is not defined as per situation a) or b), but it is not entirely absent, then it is classified as a minor disorder.

To simplify interpretation of the results, the conditions determining the presence of a positive threshold are identified with capital letters (Annex 1, paragraphs EI, E2, E3).

For the lower back, acute lumbar pain (Annex 1, paragraph E4) is reported separately. Acute lumbar pain is defined as “presence of intense lower back pain, with or without irradiation, that has caused immobility for at least 2 days, or 1 with medication.” When a worker reports more than 3 or 4 episodes in the last 12 months, it is probably not true acute lumbago, but may indicate a positive threshold for the lumbar spine.

In the section concerning the past medical history, subjects are asked if, having reported disorders in the last 12 months, they are aware of any previously diagnosed spine musculoskeletal disease (Annex 1, paragraph E5), such as, for example, a herniated disc. In this case, the subject is also asked to produce the results of the relevant instrumental examinations, confirming the reported pathology.

The spinal anamnesis ends with dos sections completed by the compiler, with remarks on possible future treatment to be recommended to the subject (Annex 1, paragraph E6), or for reporting the results for exposure level to biochemical overload, when known (Annex 1, section F).

#### Lower limbs disorders: the anamnestic investigation model

The structure of this recently added section (Annex 1, section G) comprises questions about pain affecting the hips, knees, and feet. The definition of positive threshold uses similar criteria to those adopted for the upper limbs, given that these disorders derive primarily from inflammation of tendons and joints.

#### Summary of musculoskeletal disorders, with positive threshold in the last 12 months

The last of the anamnestic questionnaire (Annex 1, section H) includes body maps, which are useful for providing a visual summary of the anamnestic examination and the joints found to be positive at the various specific anamnestic thresholds.

#### Validation testing of the anamnestic questionnaire

The inter-rater and intra-rater reproducibility of the questionnaire was previously tested by examining agreement between the results obtained from 99 questionnaires administered by the same observers and by different trained observers. This was named evaluators’ group, composed by 37 physicians from 14 European and Latin American countries: the Latin health surveillance group.^6^

#### Methods to administer the anamnestic questionnaire

It seemed useful to list below two ways of administering the anamnestic questionnaire, and each approach requires the data to be gathered differently:

- Method 1: the healthcare professional administers the anamnestic questionnaire to workers before a clinical examination;
- Method 2: the questionnaire is administered in a guided manner to groups of up to 10-15 exposed workers, under the supervision by a trained healthcare professional o nurse. This method may be employed as a means of sharing information with workers and offers an excellent opportunity para to explain the disorders (e.g. what causes them and how they can be prevented).

### PRESENCE OF MUSCULOSKELETAL DISORDERS IN WORKING POPULATIONS NOT EXPOSED TO BIOMECHANICAL OVERLOAD

Data concerning reference groups of workers not exposed to biomechanical overload were taken from the reference groups more recently analyzed: Group No. 2, with 2,015 employees,^8^ Group No. 3, with 1,046 employees,^10^ and Group No. 4, with 1,387.^9^

The results obtained with the previous reference groups^2,3,7^ were however very similar to the older data.

Table 1 shows the prevalence of work-related musculoskeletal disorders (WMSD) reported and diagnosed previously (based on instrumental examinations), for the subjects in the reference groups at the time of administration of the anamnestic questionnaire.

**Table 1:** Positive thresholds for spine, upper limbs, and lower limbs, broken down by gender and age, obtained from reference groups 2, 3 and 4

Spine: positive pain thresholds, acute lower back pain, and lumbosacral (LS) herniation										
	Cervical		Dorsal		Lumbosacral		Acute lumbago		LS hernia/protrusion	
	M	F	M	F	M	F	M	F	M	F
Age										
15-35	8.6%	22.0%	2.1%	3.3%	4.2%	12.4%	2.5%	2.4%	3.7%	2.6%
36-55	15.5%	32.5%	4.0%	7.3%	10.6%	21.5%	6.7%	5.5%	9.1%	4.7%
> 55	14.4%	26.5%	2.4%	13.6%	14.3%	43.9%	2.2%	8.2%	4.8%	15.2%
Total for gender	12.5%	27.4%	3.0%	6.2%	8.1%	18.8%	4.6%	4.4%	6.3%	4.9%
Total	20.0%		4.9%		15.0%		4.5%		5.4%	

Upper limbs: positive pain thresholds												
	Shoulder			Elbow			Wrist/hand			Nocturnal paresthesia		
	M	F	M+F	M	F	M+ F	M	F	M+F	M	F	M+F
Age												
15-35	2.3%	4.2%	3.3%	0.9%	0.2%	0.6%	0.7%	3.3%	2.0%	0.4%	3.1%	1.7%
36-55	4.5%	9.4%	7.0%	1.7%	2.2%	1.9%	1.9%	4.6%	3.0%	0.7%	10.9%	5.4%
> 55	5.9%	18.1%	10.9%	1.3%	0.0%	1.1%	1.3%	0.0%	1.1%	0.0%	17.6%	3.2%
Total	3.7%	7.5%	5.6%	1.3%	1.2%	1.3%	1.3%	3.9%	2.5%	0.6%	7.4%	3.7%

Lower limbs: positive pain thresholds for knee pain			
	Knee		
	F	M	F+M
15-35	8.4%	6.6%	7.3%
36-55	7.1%	13.5%	10.8%
> 55	19.0%	19.7%	19.4%
Total	8.9%	11.1%	10.1%

LS = lumbacral; M = male; F = female.

A good correspondence was found between the percentages of positive thresholds for traits and the percentage of certain pathologies diagnosed, except for the presence of nocturnal paresthesia, which was found to have little correlation with presence of carpal tunnel syndrome, diagnosed with electromyography. The reason is however known, since very few of the respondents, who were found to have a positive threshold for nocturnal paresthesia, did not request further clinical investigations for these disorders, not considering them worthy of further clinical investigation.

### EXAMPLES OF USE OF THE ANAMNESTIC QUESTIONNAIRE TO AN EXPOSED POPULATION COMPARED WITH RESULTS OBTAINED IN UNEXPOSED POPULATIONS (REFERENCE GROUPS): SOME EXAMPLES OF APPLICATION

#### Premise

We will describe the results of some unpublished anamnestic studies conducted with this questionnaire model by several teams of occupational health physicians.

We will report only the main data concerning the prevalence of positive cases for different anamnestic thresholds for the segments affected by biomechanical overload.

It is indeed the use of the questionnaire that makes it possible, having evaluated the type of risk for biomechanical overload, to focus the clinical investigation on only certain segments (upper limbs, or lumbosacral spine, or lower limbs, etc.). The Italian legislation establishes that periodic health surveillance should be conducted only when risk is present, aiming to achieve a qualitative and quantitative balance.

With regard to statistical evaluations conducted for the several groups, i.e., the presence of significant differences in comparison with the reference population, relative risk (RR) was used after direct standardization. Relative risk is a statistical term used to refer to the number of times an event occurs in a group in comparison to another group. It is generally sed in clinical epidemiology and evidence-based medicine to determine the relationship between the prevalence in exposed and not exposed to the same risk factor. Results for the RR should be interpreted as follows: a) if the RR is = 1, there is no association between the risk factor and the disease; b) if the RR < 1, there is an inverse association, i.e., the probability of developing the disease is lower for those exposed to the risk factor; c) if the RR > 1, there is an association, i.e., the probability of developing the disease is greater for exposed individuals.

It is important to include the study of the confidence interval (CI). Its calculation yields two values, a lower and an upper value. When the lowest CI is greater than 1, the difference between the group of exposed individuals and the reference group is certainly present.

The groups presented were analyzed separately by gender and by age group (three age groups: 15-35; 36-55; older than 55).

Exposed and reference populations were compared applying the direct standardization method, taking the unexposed population as the reference group, which allows to adjust las prevalence rates considering the difference structures of age and sex of the populations to be compared.

#### Group of carpentry workers (sanding): study of upper limbs

**Figure 1:**
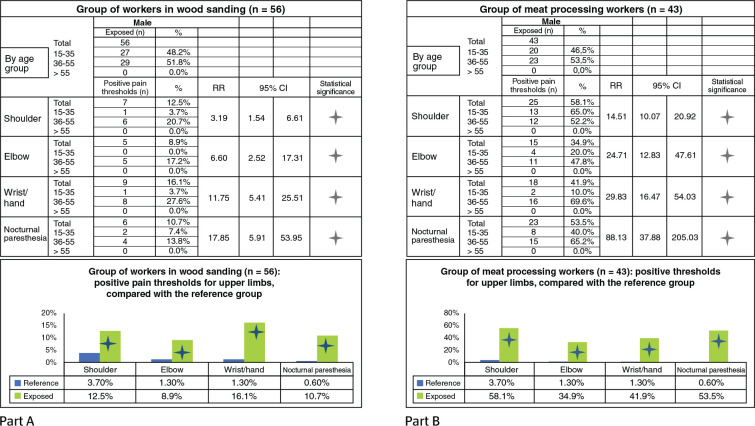
Statistical significance of the results from the anamnestic evaluation of two groups of workers: wood sanding (A) and pork processing industry (B). RR = relative risk.

The group analyzed (56 men) comprised individuals working with wood sanding, partly by hand and partly using an abrasive disc. This work implies a medium/high biomechanical overload on upper limbs, with repetitiveness of movements (medium/high frequency of action, often moderate effort, forced postures, etc.). Anamnestic thresholds for upper limbs were positive from 10% to 15% for all areas. There are significant differences regarding the reference group for all upper limb areas analyzed (Figure 1, Part A).

#### Group of pork processing workers (several cuts): study of upper limbs

The analyzed group (43 men) workers in the pork processing industry (especially in the cutting and boning operations). This work implies a very high biomechanical overload on the upper limbs, due to high repetitiveness of movements (high frequency of action, often moderate, but also strong, effort, awkward postures, etc.). The anamnestic thresholds for the upper limbs are positive from 40% to 60% of the workers; with higher values for the shoulder and nocturnal paresthesia. There are significant differences regarding the reference group in all upper limb areas analyzed (Figure 1, Part B).

#### Group of fruit packing workers: study of upper limbs

The group analyzed consists of both male (n = 310) and female (n = 180) workers in fruit packing, especially peaches, apricots, persimmons, and kiwis. This work implies upper limb biomechanical overload, medium for the male gender and high for the female gender, especially due to the high repetition of movements (high frequency of action, often moderate effort, awkward postures, especially of the shoulder, etc.).

**Figure 2:**
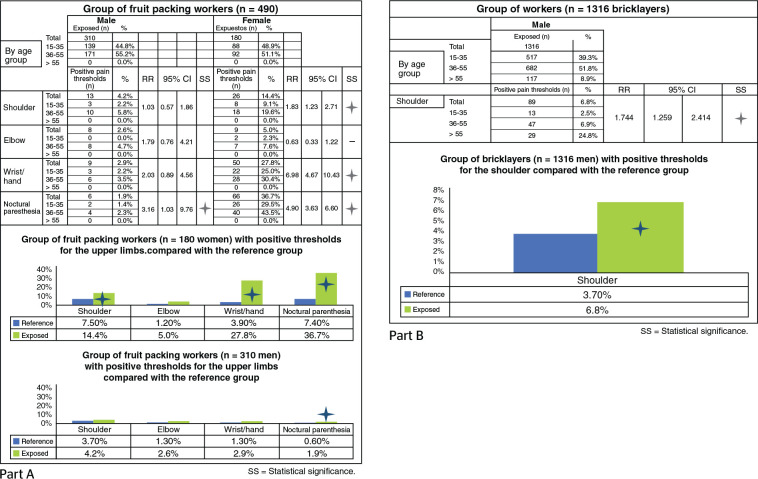
Statistical significance of the results from the anamnestic evaluation in two groups of workers: fruit packing workers (A) and bricklayers (B).

The percentage of positive anamnestic thresholds for upper limbs is 10% to 30% in women; the highest percentages were observed for wrist/hand and nocturnal paresthesia. There are significant differences with regard to the reference group for the regions of shoulder, wrist/hand, and nocturnal paresthesia (Figure 2, Part A). For the male gender, anamnestic thresholds for the upper limbs are positive only for 2% to 4% of workers, and significant differences in relation to the reference group are present only for nocturnal paresthesia.

#### Group of bricklayers: study of the shoulder

The group includes 1316 construction workers employed as bricklayers.

Currently, there are available data analyzing the prevalence of positive anamnestic threshold only for the shoulder (Figure 2, Part B). The anamnestic threshold for the shoulder is positive for 6.8% of workers (3.7% in the reference group), with a significant difference in regarding the reference group.

#### Daycare teachers: study of spine

**Figure 3:**
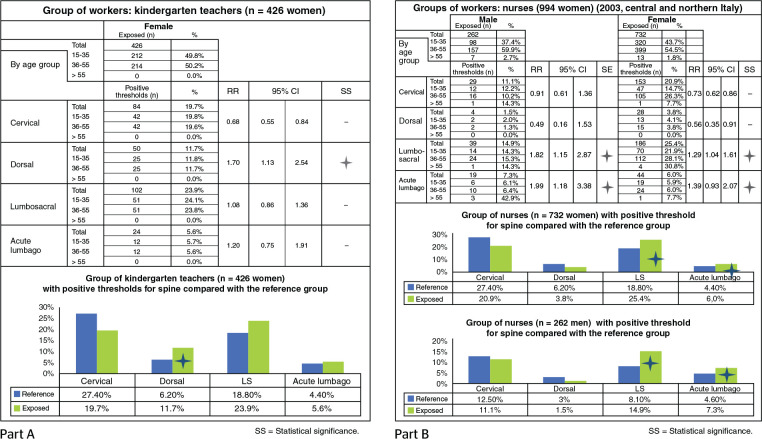
Statistical significance of results from the anamnestic evaluation of two groups of workers: kindergarten teachers (A) and hospital nurses (n = 262 male y n = 732 female) (B). RR = relative risk.

The group analyzed, which consists of workers from a number of municipalities in northern Italy (426 females), dedicated to the care of children younger than 3 years of age (daycare teachers). This work leads to different levels of biomechanical overload on spine (medium/low depending on the child to be handled), due to the frequent need to lift or carry the children, which may vary significantly according to the structure of the furniture. The percentage of positive anamnestic thresholds for spine ranges from 6% to 21%. Statistical significance are found for dorsal spine (Hold the child in the arms?); for the other segments, there is no significant difference in relation to the reference group (Figure 3, Part A).

#### Nurses in central and northern Italy 2003: study of spine

The group of nurses analyzed here comprises the personnel of many hospitals in northern and central Italy and includes 262 men and 732 women.

There is statistical significance for lumbosacral spine and for acute lumbago in the last year; no significant differences are observed for the other segments regarding the reference group (Figure 3, Part B). No significant differences are found between the two genders.

#### Nurses and hospital assistants in southern Italy: study of shoulder, lumbosacral spine, and knee

In this subsequent group of nurses and hospital assistants from several municipalities in central and southern Italy (262 men and 732 women), only the shoulder, the lumbosacral spine, and the knees were anamnesically evaluated, which confirms the suspicion of greater biomechanical overload on these anatomical segments.

**Figure 4:**
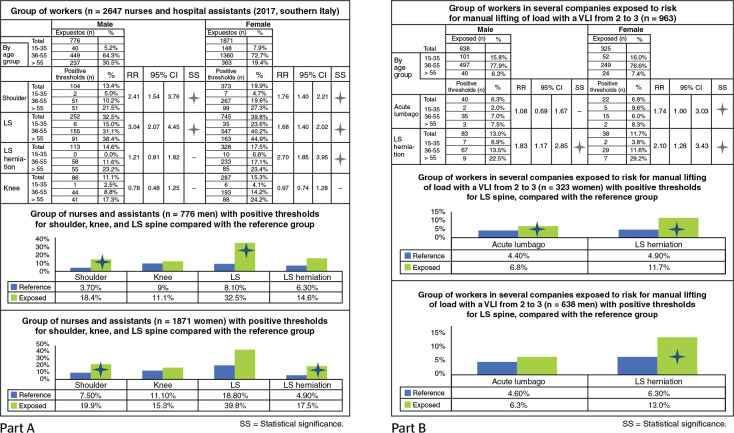
Statistical significance of the results for the anamnestic evaluation of two groups of workers: nurses and hospital assistants (A) and subjects exposed to manual lifting of loads with VLI from 2 to 3 (B). VLI = variable lifting index.

Statistical significance is observed for the shoulder and the lumbosacral spine in both sexes and for LS herniation only in women; for the knees, there are no differences in relation to the reference group (Figure 4, part A).

#### Individuals exposed to manual lifting of loads with a variable lifting index (VLI) from 2 to 3

The last group presented here consists of workers from different companies who are all exposed to risk for biomechanical overload due to manual lifting of loads, with a VLI from 2 to 3.^15^ These are average risk exposure values.

There are significant differences for herniated lumbar disc in both genders and for acute lumbago only in the female gender (Figure 4, part B).

## CONCLUSIONS

The recently updated anamnestic model of the questionnaire presented here^6^ differs from other models proposed in the literature because it employs a predetermined positive threshold that, even after collecting anamnestic data, can be used to conduct epidemiological studies, which make it possible to compare collective data on an exposed working population with those of reference populations not exposed to biochemical overload. A working group composed of 37 physicians from 14 Latin countries participated in updating and validating this model (hence named Latin Questionnaire), assessing its intraand inter-rater reliability.

This work presents several examples of application of the questionnaire, also aiming to obtain the first results of epidemiological studies, based only on anamnestic cases. These evaluations are facilitated by a software tool (Excel spreadsheets free to download from www.epmresearch.org in English, Italian, Spanish, Portuguese, and French) to allow healthcare personnel, also without a specific expertise, to conduct epidemiological studies and process the collective results by comparing exposed and unexposed populations.

The health care personnel in charge of filling in anamnestic form presented here are required to enter basic, clearly specified information into the software, such as personal data, positive thresholds, minor disorders, acute lumbago (at least one episode in the last 12 months), and disorders diagnosed previously. The software calculates the prevalence in unexposed individuals, performs direct standardization, and expresses the result as relative risk with relative confidence interval. After uploading the data from a homogenous group of workers on risk exposure, histograms comparing the data for exposed and unexposed workers and their statistical significance are plotted automatically. Therefore, occupational physicians may also benefit from a very useful results from the clinical/epidemiological investigation, already in the anamnestic phase.
